# Supplementary material for: Synthesis and Study on Ni-Co Phosphite/Activated Carbon Fabric Composited Materials with Controllable Nano-Structure for Hybrid Super-Capacitor Applications
Source: Nanomaterials (Basel). 2021 Jun 23;11(7):1649. doi: 10.3390/nano11071649 (PMC8304602; doi:10.3390/nano11071649)
Supplement: Supplementary file 1 [file nanomaterials-11-01649-s001.zip › nanomaterials-1242781-SI.pdf]

Supplement materials

Synthesis and Study on Ni-Co Phosphite/Activated Carbon Fabric Composited Materials with Controllable Nano-Structure for Hybrid Super-Capacitor Applications

Dalai Jin[1,2], Jiamin Zhou [2], Tianpeng Yang [2], Saisai Li [2], Lina Wang [1], Yurong Cai [2] and Longcheng Wang [2,*]

[1] Key Laboratory of Advanced Textile Materials and Manufacturing Technology, Ministry of Education, Zhejiang Sci-Tech University, Xiasha Town, Hangzhou 310018, China; lnwang@zstu.edu.cn
[2] School of Materials Science and Engineering, Zhejiang Sci-Tech University, Xiasha Town, Hangzhou 310018, China; zhoujiamin321088@163.com (J.Z.); 729099790@qq.com (T.Y.); 2390179283@qq.com (S.L.); caiyr@zstu.edu.cn (Y.C.)
* Correspondence: wlongcheng@zstu.edu.cn

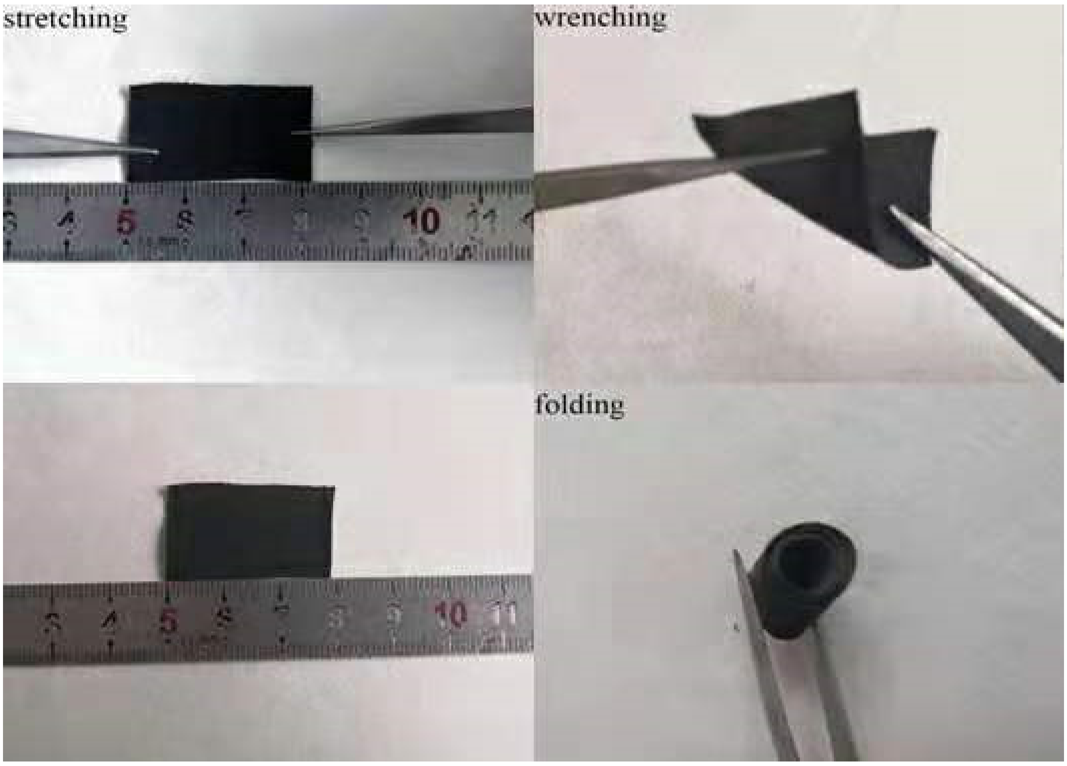

Figure S1. Digital images of the MHP/ACT composite under stretching, wrenching and folding mode.
